# Effect of Cutting Conditions on the Size of Dust Particles Generated During Drilling of Carbon Fiber Reinforced Composite Systems

**DOI:** 10.3390/polym17101323

**Published:** 2025-05-13

**Authors:** Tomáš Knápek, Štěpánka Dvořáčková, Dora Kroisová, Martin Váňa

**Affiliations:** Department of Machining, Assembly and Engineering Metrology, Faculty of Mechanical Engineering, Technical University of Liberec, Studentská 1402/2, 461 17 Liberec, Czech Republic; stepanka.dvorackova@tul.cz (Š.D.);

**Keywords:** CFRP, drilling, optical microscopy, electron microscopy, CT tomography, dust particles, respiratory hazard, delamination

## Abstract

The influence of machining parameters on the generation of dust particles during the machining of carbon fiber-reinforced polymer (CFRP) composites remains insufficiently understood. These particles, which stay suspended in the air, pose a serious health risk to operators. This study examined the effects of cutting conditions—specifically cutting speed, feed per tooth, and depth of cut—and the impact of delaminations formed during CFRP drilling on the size, shape, and quantity of hazardous dust particles. Experiments were conducted using a commercially available uncoated cemented carbide cutting tool. The analysis of dust particle size and morphology, as well as the evaluation of delamination, was performed using microscopic and tomographic methods. The results demonstrate that reducing the cutting speed led to a decrease in particle size for the investigated CFRP material. Furthermore, it was observed that tool wear results in the generation of smaller particles. Simultaneous delamination during drilling was found to significantly affect the structural integrity of the composite material.

## 1. Introduction

Composite systems reinforced with carbon fibers embedded in an epoxy matrix are widely used in technical practice. Although components are often manufactured “to measure”, finishing operations such as drilling are frequently necessary to enable joining of individual parts. During the machining of fiber-reinforced plastics (FRPs), particularly CFRP, carbon fibers easily break into fine dust particles. These particles enter the surrounding atmosphere and pose a risk to human health. Typical health problems include irritation of the eyes and upper respiratory tract, dizziness, drowsiness, nausea, and vomiting [1].

According to Kwan [2], almost all inhaled particles with an aerodynamic diameter greater than 10 µm are trapped in the nasal cavity. Particles between 5 and 10 µm settle in the nasopharynx region, while about 40% of particles sized 2 to 5 µm are deposited between the trachea and terminal bronchi. Smaller particles, with diameters below 2 µm, are capable of reaching the alveolar region of the lungs, where they can cause the most significant health damage.

Only a few studies in the literature have addressed this problem. Iyer [1] recommended protective measures when machining CFRP, based on studying dust extraction efficiency. That study referred to aerosol standards defined in EN 481 concerning workplace safety. Particles between 5 and 10 µm can irritate the nose and throat, while particles between 3 and 5 µm can reach the trachea. Particles of 1 to 2 µm in size have the potential to enter the lungs through the bloodstream. Particles smaller than 1 µm can penetrate into the alveoli and even enter the circulatory system. Therefore, the use of protective equipment, including dust masks, goggles, gloves, and protective clothing with elastic cuffs, is highly recommended. Additionally, the use of special vacuum systems with high-efficiency filters is advised to prevent short circuits caused by conductive carbon fibers.

A study by Haddad et al. [3] confirmed that machining CFRP generates chips that break down into dust particles ranging from 0.25 to 1 µm. These small particles can cause multiple health issues and often exceed occupational exposure limits. High-speed trimming was found to produce two to eight times more dust. Further experiments by Haddad et al. confirmed that machining parameters significantly influence the formation of very fine dust particles, with up to 95% of the dust capable of reaching the pulmonary alveoli.

Ramulu and Kramlich [4] observed that machining FRPs produces dust and powders consisting of fibers and cured matrix, both hazardous to health. Elgnemi et al. [5] noted that increasing the chip thickness by reducing the spindle speed or increasing the feed rate can lower dust emissions. Despite the serious impact of fine dust particles on health, their dangers are still not fully addressed. Small dust particles are able to reach the deepest parts of the respiratory system, causing serious health issues. Therefore, strict protective measures should be taken to safeguard both human health and machinery [6].

Low cutting speed combined with a high feed rate can reduce dust particle generation but may negatively affect hole quality. Thus, a compromise between cutting conditions and the quality of drilled holes must be achieved.

Many studies have since expanded on this issue, although differences in materials, cutting tools, and machining conditions make comparisons difficult [7,8]. Drilling remains the most common operation for assembling CFRP parts using bolts or rivets. For example, more than 100,000 holes are drilled in a small single-engine aircraft, and up to one million holes in a large airliner.

However, CFRP drilling is associated with defects such as peel-up and push-out delamination, fiber/resin pull-out, fiber crushing, and high surface roughness [9,10,11]. Research continues to focus on minimizing both internal damage and dust generation [12,13,14,15,16]. Dust particles consist mainly of fragmented epoxy resin and carbon fibers, with sizes ranging from hundreds of nanometers to hundreds of micrometers. These particles can remain airborne for weeks, which greatly increases their potential impact.

The present study investigated the influence of cutting conditions and the resulting delamination on the size, shape, and quantity of hazardous dust particles generated during CFRP drilling. Experiments were conducted under laboratory conditions similar to those in small-scale manufacturing. The aim was to evaluate the relationship between cutting parameters, delamination of CFRP components, and the generation of harmful particles.

## 2. Materials and Methods

This section describes in detail the machinery and equipment used to perform the experiments, specifies the characteristics of the cutting tools and material used, and presents the methodology for measuring and evaluating the experimental results.

### 2.1. Machined Composite Material

**Figure 1:**
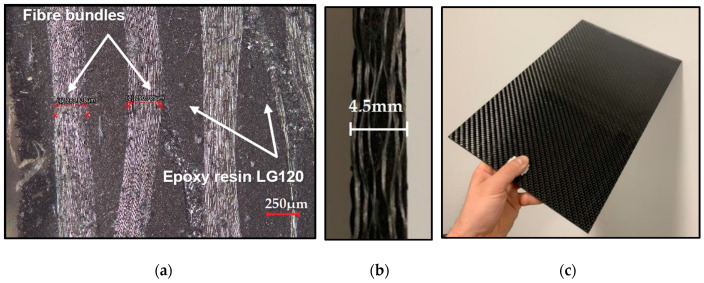
Machined composite material: cross-section of a plate of composite material at a scale of 250 μm (**a**); slab cut (**b**); composite material plate (**c**).

The composite material used for this experiment was carbon fiber-reinforced polymer. The CFRP laminate was produced using prepregs, the diameter of carbon fiber 8 μm, and a fiber volume fraction of about 60% (Kordcarbon, Strážnice, Czech Republic). The laminate was produced by vacuum infusion, where the basis was CCh600 type reinforcement (Kordcarbon, Strážnice, Czech Republic) and LG120 type matrix as the epoxy resin (Compositesplaza, B.V., Helmond, The Netherlands). The mentioned resin belongs to the low-viscosity standard lamination resins with high adhesion and good temperature resistance. Technical parameters and images of the composite board are shown in Table 1 and Figure 1. It is a material used to produce some primary and secondary aircraft parts (manufactured by a Czech company). The laminates were cut into test samples of size 300 mm × 150 mm × 4.5 mm (length × width × thickness of the sample).

### 2.2. Cutting Tool, Cutting Parameters

**Figure 2:**
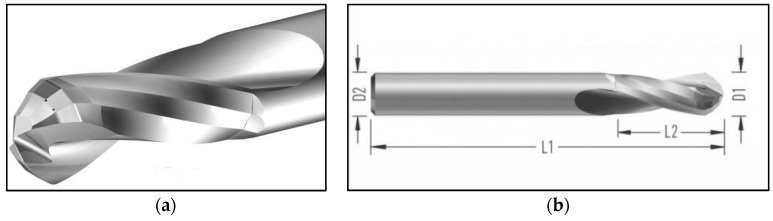
Cutting tool; (**a**) cutting tool drill tip; (**b**) drill dimensions.

A drill with a diameter of 6 mm and a working length of 12 mm was chosen as the cutting tool. The tool was made of uncoated cemented carbide. The geometry of the drill was specially designed for machining composite parts. The cutting tool was chosen because of its availability and suitability for machining CFRP laminate. Three identical drills (each for different cutting conditions) were used in this study. Cutting tool wear was measured on the back of the cutting tool. All the technical specifications of the cutting tool needed for the experiment are shown in Table 2 and Figure 2. Three levels of cutting speed *v_c_* (15, 35, and 55 m/min) were chosen, and one feed level per revolution *f_n_* (0.1 mm/rev) was used to study the effect of cutting parameters on the harmful dust generated. Drilling was performed without the use of process fluid (dry machining) to simulate a real industrial process. CFRP laminate samples were prepared for each cutting condition and drilling cutting tool. For each sample, the direction of drilling the hole was always followed according to the direction of the binding of the carbon fabric.

### 2.3. Machine Tool

**Figure 3:**
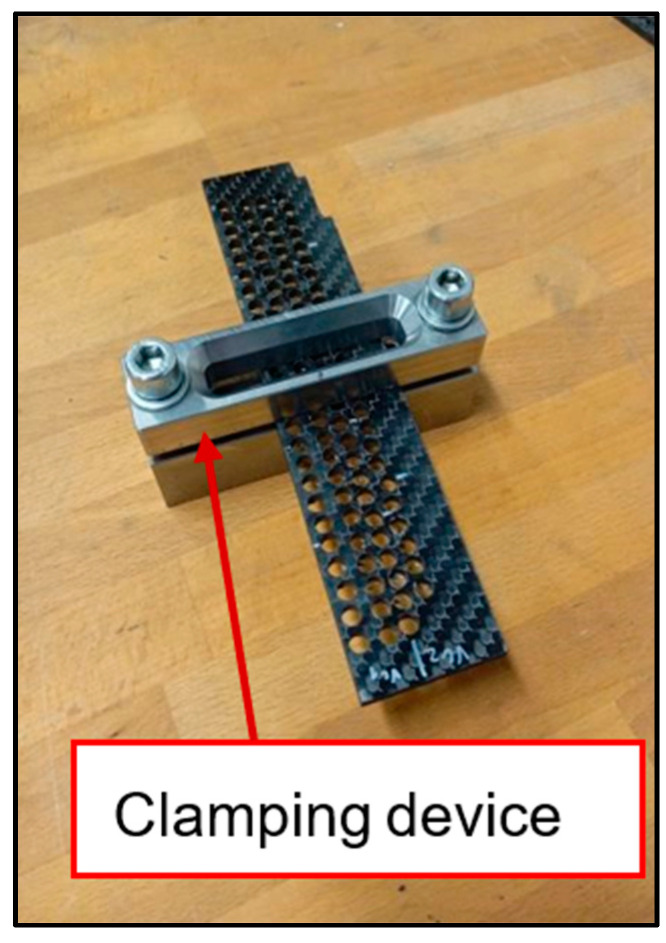
Clamping device for clamping the sample and ensuring repeatability of the drilling conditions.

The process of drilling into the CFRP laminate was completely carried out on a conventional tool milling machine FNG 32 (TOS Olomouc, Olomouc, Czech Republic) with a maximum spindle speed capacity of up to 4000 rpm, commonly used in industrial practice. The solid material was drilled without process fluid, as mentioned above. The CFRP laminate specimens were securely fixed with cap screws to a fabricated fixture commonly used for drilling and milling composite materials—Figure 3.

### 2.4. Suction Device

The dust generated during machining was extracted using a device labeled POC9 M1 (Vzduchotechik, Chrastava, Czech Republic) commonly used in laboratories and in small machine operations, the parameters of which are listed in Table 3. This commercial device is normally connected as an extraction device when drilling samples. In order to assess the size of the dust particles, non-woven fabric filters—Table 4—were inserted into part of the suction opening to trap the particles.

A special attachment, custom-made for the study’s needs, was used to extract dust particles from the cut site.

### 2.5. Collection and Analysis of Dust Particles

Harmful dust particles generated during the drilling process were weighed and analyzed from the filter of the extraction device, which had a diameter of 100 mm. A unique filter made of non-woven fabric was used for the experiment to capture dust particles, the parameters of which are listed in Table 4.

After each drilling test, the resulting harmful dust particles were analyzed using a TESCAN Mira 3 scanning electron microscope (TESCAN Holding, Brno, Czech Republic), referred to as SEM, and a Keyence VK-X 1000 confocal microscope (Keyence, Itasca, IL, USA), referred to as a confocal microscope. A confocal microscope was used to obtain 3D images to evaluate different sizes and shapes of dust particles and further assess cutting tool wear (geometric measurements of cutting edges). A ZEISS DV4 (Carl Zeiss AG, Jena, Germany) stereomicroscope was used for previews.

The electron microscope was used to monitor sub-micrometer fiber lengths, assess the nature of fiber fractures, and describe the nanometer and sub-micrometer particles produced during the drilling. The surface of the samples of tiny particles/drilled holes was sputtered with a layer of Pt-Pd (Quorum Q 150R ES) with a thickness of 2 to 4 nm. The accelerating voltage was 5.0 kV for tiny particles and reflected/scattered electrons (SE) were detected. The accelerating voltage was 10.0 kV for drilled holes and reflected/scattered/backscattered electrons (SE(BSE)) were detected.

For the statistical evaluation of the experimental data, standard deviation analysis and linear regression methods were employed. Each measured parameter was recorded three to five times to ensure sufficient data for meaningful statistical analysis.

The dust particles captured on the filter during the drilling process were analyzed using a calibrated laboratory balance T-SCALE Electronics MFG–NHB-1500+. This is a very accurate laboratory scale up to 1500 g with a weighing accuracy of 0.01 g.

A total of 50 holes were drilled with each new cutting tool. Samples of dust particles were collected after every 5th drilled hole. Before each drilling process, the machined CFRP laminate and the new filter were always considered first. Subsequently, five holes were drilled. After drilling five holes, the machined CFRP laminate and the filter were weighed. The weight of the machined CFRP and filter before and after drilling was compared. After weighing, the non-woven fabric filter was further analyzed for the shape and size of harmful dust particles by SEM and confocal microscope.

### 2.6. Cutting Tool Wear

For comprehensive research, it was also necessary to measure the wear of the cutting tool, as the tool is worn out by the abrasion of carbon fibers during the drilling process. According to several studies published so far, this wear significantly influences the occurrence of delamination itself. Analysis/measurement of the amount of cutting tool wear and delamination was performed using a confocal microscope. Measurements were made after every five drilled holes. The measurement interval was determined after drilling 1, 5, 10, 15, 20, 25, 30, 35, 40, 45 and 50 holes. As part of the wear of the cutting tool, the amount of wear on the spine was measured.

From the point of view of CFRP delamination analysis, the size of the delamination was measured for each hole, both at the entrance of the hole and at its exit. To evaluate the measured results, it was necessary to determine the limit value of the average F*_Dcrit_* delamination size at the entrance and exit from the drilled hole. There is no industry standard or prescriptive norm for a given limit value. In this study, the limit average value F*_Dcrit_* = 1500 µm was selected.

### 2.7. Delamination Analysis

From the point of view of CFRP delamination analysis, the size of the delamination was measured for each hole both at the entrance of the hole and at its exit. To evaluate the measured results, it was necessary to determine the limit value of the average F*_Dcrit_* delamination size at the entrance and exit from the drilled hole. There is no industry standard or prescriptive norm for a given limit value. In this study, the limit average value F*_Dcrit_* = 1500 µm was selected.

Pull-out, push-down delamination was measured on a confocal microscope. Delamination in the volume of the sample, namely, delamination in the layers of the composite system, was imaged using computed tomography with a ZEISS Metrotom 1500 tomograph under the following display conditions: voltage 179 kV, current 285 μA, integration time 2000 ms, gain 8.0×, binning 2 × 2, rotation speed 10.0°/s, magnification 8.47. The samples were placed on the rotary table in a standard way and visualization and data processing for CT were performed in a standard way.

## 3. Results and Discussion

This study focused on analyzing the size, shape, and frequency of harmful dust particles from an exhaust filter generated at different drilling speeds, cemented carbide cutting tool wear, and the resulting delamination.

This study was divided into two main parts. The first part focused on the effect of changing cutting conditions (*v_c_* = 15, 35, 55 m/min, *f_n_* = 0.1 mm/rev.) on the size, shape, and frequency of harmful dust particles. The second part focused on the effect of the wear of the cutting tool with the proportion of CFRP delamination on the size, shape, and frequency of generated dust particles from the filter of the extraction device.

### 3.1. Effect of Cutting Conditions on the Size of Dust Particles

Table 5 shows the average values of the frequency of individual sizes of dust particles on the filter for the intervals of drilled holes. Particles were detected using confocal microscope and SEM images.

The percentage of particles smaller than ten μm in size was monitored during the analysis of harmful dust particles. This is the limit value for inhaling particles that are harmful to human health.

From the collected dust particles, it was found that for the cutting speed *v_c_* = 15 m/min, the proportion of particles smaller than five μm was 51%. For the cutting speeds *v_c_* = 35 and 55 m/min, it was 58%. The analysis shows that an increase in the cutting speed promotes an increase in the amount of harmful dust particles smaller than five μm in size. Cutting speeds *v_c_* = 35 and 55 m/min generate more dangerous dust particles, which are more harmful to human health.

**Figure 4:**
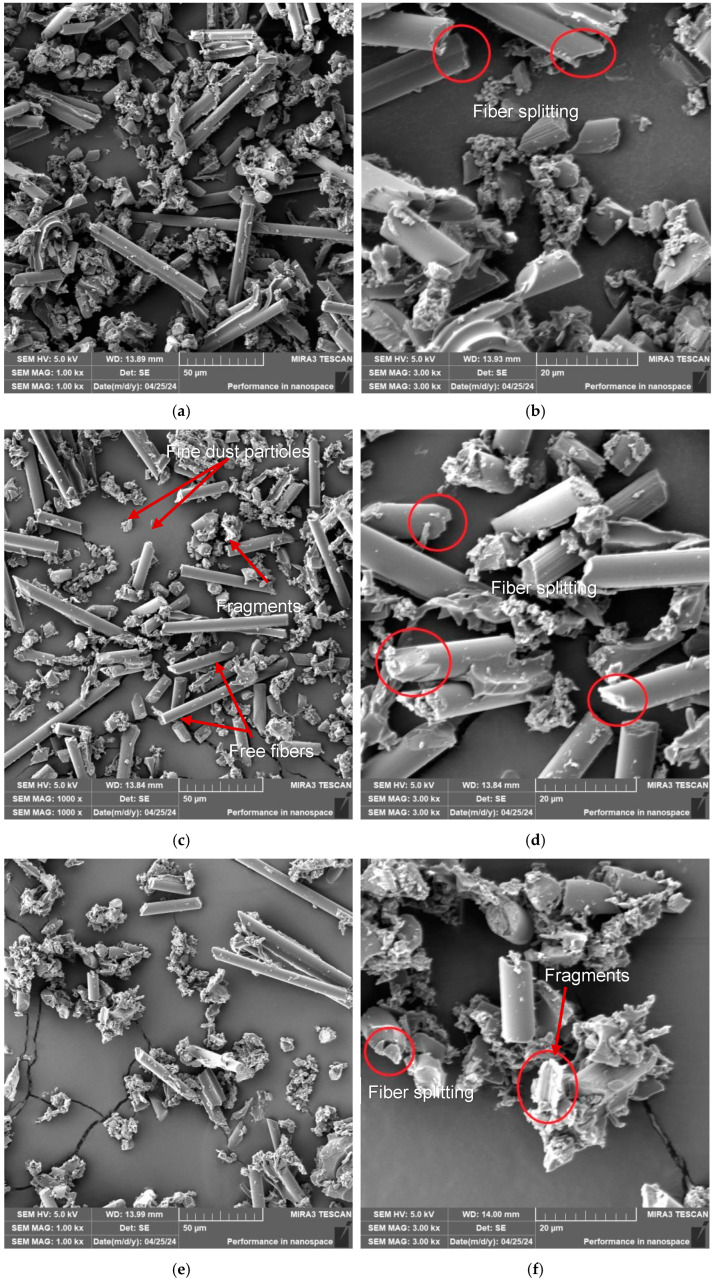
SEM analysis—dust particles captured on the filter; (**a**) v_c_ = 15 m/min, f_n_ = 0.1 mm/ot, (**b**) zoom on (**a**); (**c**) v_c_ =35 m/min, f_n_ =0.1 mm/ot, (**d**) zoom on (**c**); (**e**) v_c_ = 55 m/min, f_n_ =0.1 mm/ot, (**f**) zoom on (**e**).

The SEM analysis shown in Figure 4 found that the carbon fibers broke at an angle or perpendicular to the longitudinal axis of the fibers. No longitudinal fiber breaks were evident. The fibers had a diameter of 6 μm, and their length ranged from units to tens of micrometers. Carbon fibers were split transversely or at 40–60° angles, i.e., with a pointed end. In addition, they also chipped off at the ends, creating the smallest dust particles. Despite the temperature that is generated when drilling the holes, there is no thermal effect on the shape and length of the particle characters. Epoxy resin, as a reactive plastic matrix, fractures brittlely and small fragments tend to aggregate. The emerging temperature does not affect the refractive character of the thermally conductive carbon fibers.

From a detailed analysis of the dust particles captured on the filter using a confocal microscope and SEM, the presence of four types of dust particles was proven:

fine dust (mixture of matrix and fibers) with dimensions from 1 μm to 5 μm,

free fibers with dimensions from 10 μm to 400 μm,

matrix pieces with dimensions from 5 μm to 40 μm,

fragments (matrix + fibers) measuring 100 μm to 500 μm.

From the results in Table 6, as the cutting speed increases, the size of the dust particles decreases, and the frequency of the generated dust particles increases. The most significant amount of generated chips of the most diminutive dimensions was created at a cutting speed of *v_c_* = 55 m/min. On the contrary, the smallest particles were made at a cutting speed of *v_c_* = 15 m/min. At the same time, at the highest cutting speed, *v_c_* = 55 m/min, the average size of the dust particles was 7.30 μm, while at the lowest cutting speed, *v_c_* = 15 m/min, the average size of dust particles was 9.01 μm. 

Elevated cutting speeds during CFRP machining significantly affect dust particle generation by enhancing brittle fracture behavior in both carbon fibers and the polymer matrix. The associated increase in cutting temperature reduces the mechanical strength of the matrix, promoting micro-fracturing and particle detachment. Moreover, higher cutting speeds concentrate the mechanical and thermal energy within a smaller interaction zone, resulting in intensified material disintegration. Consequently, higher cutting speeds lead to the formation of a greater number of ultrafine dust particles, which increases occupational health risks and necessitates effective dust extraction and filtration systems.

**Figure 5:**
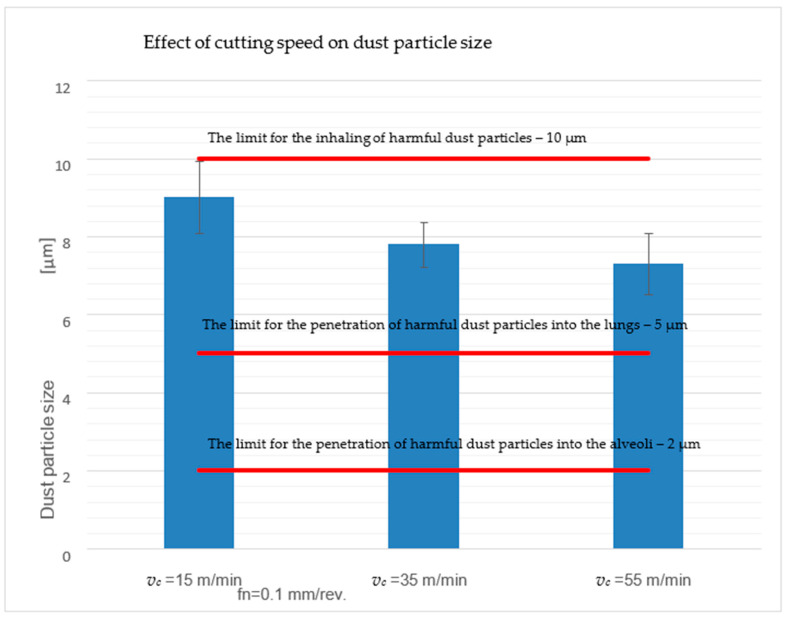
Effect of cutting speed on the size and frequency of dust particles.

The measurements performed show that the size and frequency of dust particles are significantly influenced by the size of the cutting speed (Figure 5). As the cutting speed increased from 15 to 55 m/min, smaller dust particles formed, and their frequency increased.

This means that a combination of a high cutting speed of 55 m/min and a feed of 0.1 mm/rev creates small, tiny, hazardous harmful dust particles (5 and 10 µm settle in the area of the nasopharynx or larynx, 2 and 5 µm settle between the trachea and bronchi, and particles smaller than 2 µm can then reach the bloodstream) [1,2].

As part of the study, the effect of the cutting speed on the amount of captured dust particles on the filter was also monitored. Dust particles caught on the extraction device’s filter and the machined CFRP were weighed on a calibrated laboratory scale, T-SCALE Electronics MFG—NHB-1500+. The dust particles caught on the extraction device’s filter and the machined material were weighed, and the percentage ratios, which are listed in Table 7, were then calculated from these values.

As the cutting speed increases, the suction device’s efficiency increases noticeably. At the lowest cutting speed, *v_c_* = 15 m/min, the average ratio of extracted dust was only 26.1%. The rest of the chips remained trapped in the jig, on the machine plate, and scattered in the air. In contrast, at the highest cutting speed, *v_c_* = 55 m/min, the average ratio of extracted dust was up to 92.5%, i.e., a difference of up to 66.4%.

This phenomenon occurs because, at a higher cutting speed, the tool rotates so fast that the dust particles are more dispersed into the air, and thus, the extraction system can extract them better. At a low cutting speed, the chips tend to fall into the fixture onto the machine plate, and they are not dispersed into the air as much.

### 3.2. Effect of Cutting Tool Wear on the Size of Dust Particles and the Issue of Delamination

**Figure 6:**
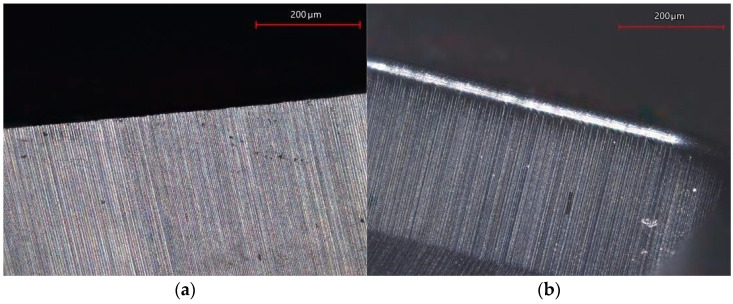
Detail of the tool edge; (**a**) new tool edge; (**b**) worn tool edge.

The wear values of the cutting tool were measured after 50 drilled holes. It follows from the performed measurement that as the cutting speed increases, so does the value of the wear of the cutting tool, Table 8. The wear of the cutting tool was realized in the form of abrasion following Kroisova et al. [17,18], caused by abrasive carbon fibers acting on the tool as a polishing medium. The chips acted on the back of the cutting tool, resulting in a smooth and rounded area above the cutting edge, Figure 6. On the surface of the cutting tool, tiny grooves and small local potholes were always formed, which were caused by the bending and deflection mechanism of the carbon fiber from the cutting point [6,8].

#### 3.2.1. Effect of Cutting Tool Wear on the Frequency of Generated Harmful Dust Particles

The measurements clearly show that as the number of drilled holes increases, i.e., the tool wear increases, the frequency of harmful dust particles with a size of 1–5 μm also increases (Table 9).

As the tool wears, the frequency of dust particles with a size of 1–5 μm increases by more than twofold. The matrix and the fibers were crushed into tiny pieces and mixed to form a finer dust dangerous to human health. It was shown that the wear of the cutting tool affected the frequency of hazardous dust particles. While the new, unworn tool tended to cut the fibers, the worn edge bent, broke, and crushed the fibers into finer dust that is very dangerous to human health.

#### 3.2.2. Issues of Sample Delamination at the Entrance/Exit and Inside the Drilled Hole

Due to the negative effect of delamination on the structural quality, especially the integrity of laminates, and on the shape, size, and frequency of dust particles, this study also focused on this influencing factor.

**Figure 7:**
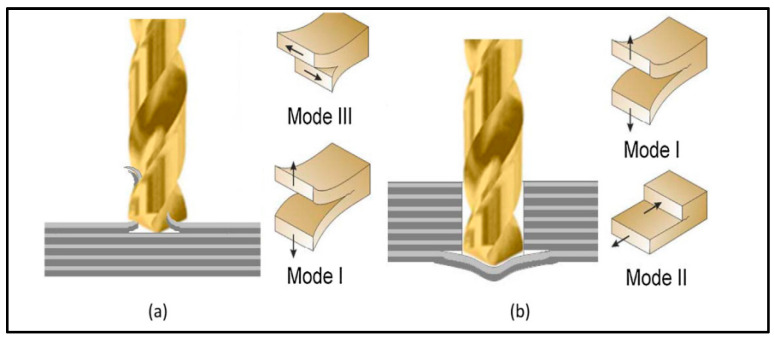
Mechanism of delamination of composite material during drilling; (**b**) delamination on the input; (**a**) delamination on the output [8].

Delamination is commonly defined as the separation of composite plies by the formation of interlaminar cracks. It is the most dominant machining defect in the drilling of thermoset CFRP, as it is often considered a limiting factor for machining tolerances and the reliability of drilled CFRP parts. Delamination occurs at both the tool entry and exit sides of the drilled hole. At the entry side, delamination happens due to peeling up the fibers as a result of a peeling force applied in an upright direction, which causes the debonding of either single fibers or the whole upper ply. However, the more severe damage happens on the exit side. There, the drill pushes the last remaining plies downward when the drilling process is getting to its end. Under this applied deformation, the thrust force may exceed the interlaminar bond strength of the laminate, leading to a separation of plies. Hence, this kind of delamination is called push-down delamination. The model of the delamination mechanism is shown in detail in Figure 7 [7,19].

**Figure 8:**
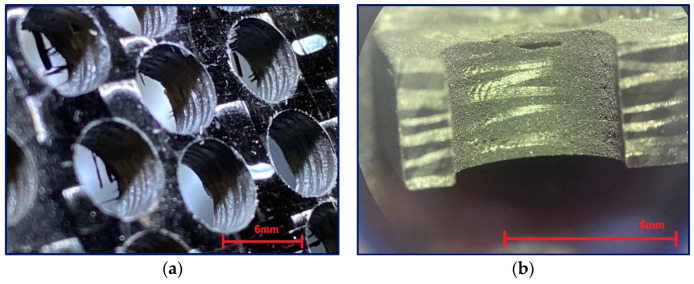
Optical microscopic images of drilled holes; (**a**) a set of holes with visible delamination at the hole entrance/exit; (**b**) a close-up of a drilled hole with clearly visible delamination at the exit.

**Figure 9:**
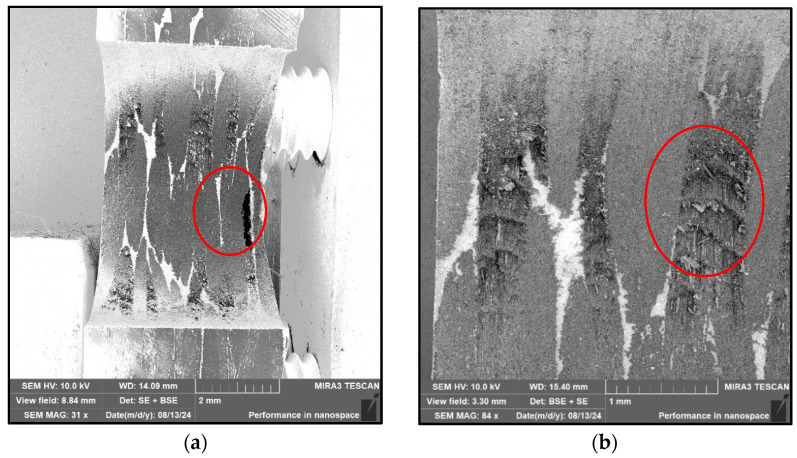
SEM microscopic images of a drilled hole; (**a**) overview image of a hole with visible push-down delamination; (**b**) a close-up of the drilled hole inside with visible destruction of the reinforcing carbon fibers.

**Figure 10:**
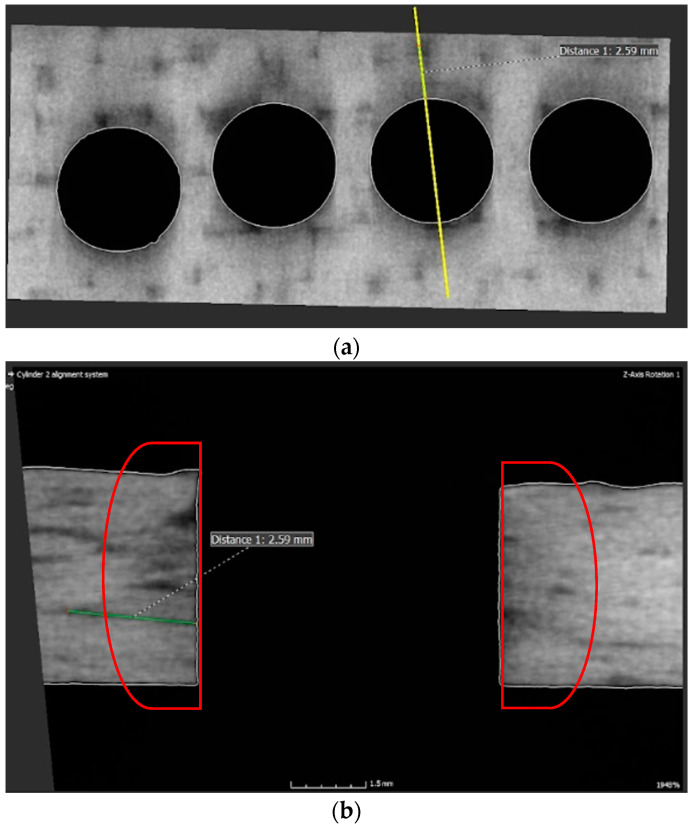
CT images of drilled holes from computed tomography. The evaluated sample (yellow highlighted cut point) (**a**) with a visible area of delamination. Display of delamination inside a hole, indicating the extent of material damage due to delamination (**b**).

The delamination of the evaluated samples caused by drilling was monitored during the study using a stereomicroscope (Figure 8, SEM Figure 9) and computed tomography (CT—Figure 10).

Figure 8 shows pull-out, push-down, and inside-the-hole delamination. Delamination is a very complex problem that includes the mechanics of delamination, the quality of the bond between the reinforcing fibers and the matrix, and the influence of the textile bond [7,18,19]. During drilling, heterogeneous material (fibers, matrix) is removed.

The removed material is ground during drilling. The fibers and the matrix are crushed, affecting the character of the surface inside the hole. The character of the fibers is clearly visible in the images from the electron microscope (Figure 4). They are shortened, broken, and chipped at the ends. This crushed material, generated during drilling, further destroys the material inside the holes.

**Figure 11:**
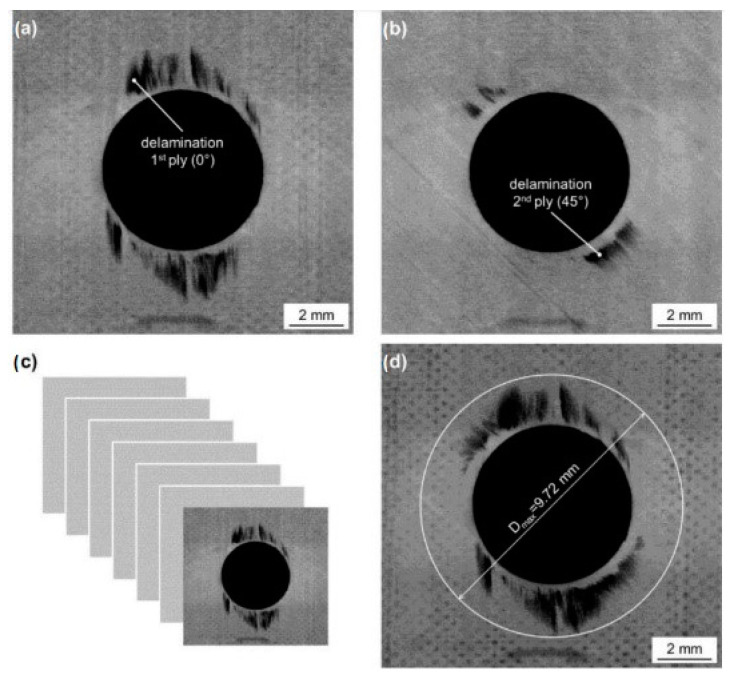
CT images of drilled holes with different levels of delamination to varying drilled hole depths [20].

The obtained results, Figure 9 and Figure 10, are in accordance with Figure 11 [20] about the occurrence of different levels of delamination in individual layers of the composite system.

As is evident, the issue of drilling composite materials is very complicated and includes many parameters that interact with each other. As shown in Figure 7 [7,19], multiple types of delamination occur when drilling a multilayer composite system. Peel-up delamination on the surface is created by pulling the fibers upward as the drill bit passes through the composite system. The threads will break and create an unwanted burr on the surface of the hole.

When the drill bit passes through the material, the resin matrix is crushed, and the fibers are cut, broken, and pulled out according to how they are placed in the direction of the drill bit movement. The fibers released in this way simultaneously enter the space between the drill bit and the wall of the material and are further ground and crushed. Due to the mechanical parameters of carbon fibers, this crushed material can have a very destructive effect on the used drill, whose blade becomes dull. Chipping of submicrometer/nanometer particles from the ends of the carbon fibers is likely to occur at this stage. These particles, on the one hand, stick to the inner surface of the holes, and on the other hand, they are sucked out during drilling. This study confirmed previous research results that the greatest delamination occurs in the middle of the thickness of the evaluated sample [20]. This fact is probably correlated with the distribution of the carbon reinforcing fibers, the highest stiffness of the system, and the highest energy that the drill has to develop to pass through the material. Delamination in this central part of the sample, Figure 10, is then the cause of reduced integrity around the drilled hole or the integrity and stability of the entire system. It can be seen from Figure 10 that the delamination between the layers detected by CT analysis reaches a length of 2.59 mm. The thickness of the sample is 4.5 mm.

When the drill bit passes through the lower part of the composite material, the fibers are pushed out on one side and separated from the matrix. The tensile load of the fibers increases, causing their subsequent destruction. This delamination is called push-down and is characterized by the simultaneous creation of a cavity between the final and penultimate layers of the composite system (Figure 10 and Figure 11). After passing the drill, the fibers break due to their tensile strength limit being exceeded and create visible burrs on the lower surface of the sample, which makes it difficult to perform other technological operations (Figure 10).

**Figure 12:**
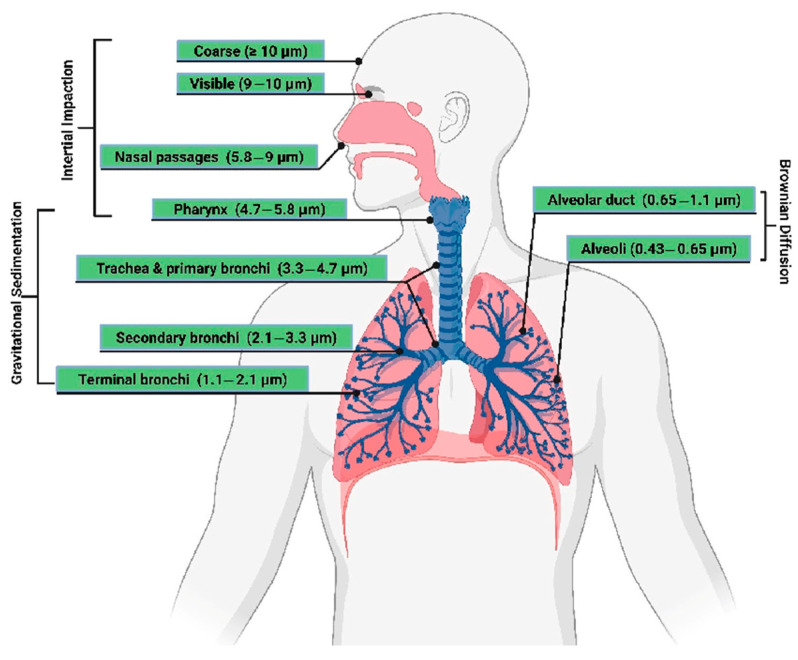
Model representation of particles that can enter the respiratory tract of the human organism [21].

From the above analysis, it is clear that the drill’s passage through the central part of the system has the greatest impact on the formation of micrometer/submicrometer and nanometer particles dangerous to human health [21] (Figure 12). This part is also the most problematic in terms of reducing the integrity of the material caused by the failure of the composite system’s cohesion layers (Figure 7, Figure 8 and Figure 9). The presence of fine particles is all the more dangerous the smaller and lighter they are, as they can then move in the air for longer periods of days or weeks and can be transported over distances of hundreds of kilometers. Based on these facts, it is clear how essential it is to incorporate filtration equipment into plant machining composite systems, particularly with carbon fiber, to minimize the release of these particles into the environment and maximize human protection. Even the simplest extraction equipment that is co-joined with the drilling area of composite systems will provide at least partial protection for the operator. There may also be opportunities to minimize dust particles and delamination in new equipment and new processes [12,13,14].

The issue of studying the drilling of composite systems is interesting both from the point of view of setting optimal cutting conditions and minimizing tool wear, as well as from the point of view of minimizing the formation of dust particles during machining. No less important is the study of the possibilities of influencing the delamination process by setting the technological conditions and any subsequent operation, which could minimize or eliminate the resulting defects.

## 4. Conclusions

The conducted study revealed several important findings regarding the generation of hazardous dust particles during CFRP drilling and their dependence on cutting conditions and tool wear. It was confirmed that increasing the cutting speed leads to the formation of smaller dust particles while simultaneously increasing their total number—particularly in the critical 1–5 μm size range, which poses the greatest health risks. A cutting speed of 55 m/min is recommended, as it offers a favorable compromise between reducing particle size and maintaining drilled hole quality. However, this speed must be paired with a high-efficiency extraction and filtration system to effectively capture the elevated quantity of finer airborne particles. Filtration systems capable of capturing ≥99.9% of particles ≥0.3 μm are considered essential for mitigating occupational exposure risks.

Tool wear was identified as a major contributor to hazardous dust formation. As the cutting edge degrades, the number of respirable dust particles increases substantially, especially in the 1–5 μm range. It is therefore advisable to replace the cutting tool after a defined number of drilled holes to limit dust emission, though the exact replacement interval must be tailored to the specific cutting tool and machining conditions.

This study also revealed that internal delamination along fiber layers leads to additional particle generation due to fiber fracture, chipping, and crushing. Minimizing delamination is thus critical not only for maintaining the mechanical integrity of the drilled component but also for reducing the airborne particle concentration. Careful optimization of drilling parameters is necessary to mitigate this dual impact.

These findings underline the importance for manufacturers to actively adjust machining strategies, monitor the tool condition, and invest in effective dust extraction solutions. Personal protective equipment and well-sealed filtration systems should be standard in CFRP machining environments to ensure operator safety.

This study was limited to one type of cutting tool and composite material, tested under controlled laboratory conditions. Future research should focus on the influence of various tool geometries, coatings, and advanced cooling or lubrication strategies on particle generation. Further work is also needed to establish precise tool life thresholds and evaluate dust control technologies in real industrial settings.

## Figures and Tables

**Table 1:** Parameters of the machined composite material.

Parameter	Value
Thickness (mm)	4.5
Width (mm)	42
Length (mm)	250
Method of production	Vacuum infusion
Matrix	Epoxy resin LG120
Hardener	HG 356
Reinforcement	CCh600
Reinforcement grammage (g/cm^2^)	600
Binding type	twill 2 × 2
Direction of fibers	0°/90°

**Table 2:** Parameters of the cutting tool.

Parameter	Value
Diameter D1 (mm)	6
Length of the cutting part of the drill bit L2 (mm)	12
Total drill length L1 (mm)	57
Diameter of the clamping part of the drill D2 (mm)	6
Number of blades	2
Cutting material	Sintered carbide
Coating	No coating
Manufacturer’s designation	V6010 UC-12594

**Table 3:** Parameters of the suction device POC9 M1.

Parameter	Value
Suction pressure (Pa)	900
Extracted air quantity (m^3^/h)	1200
Electric motor power (kW)	0.7
Loudness (dB)	65
Minimum particle capture size (µm)	0.3

**Table 4:** Nonwoven fabric parameters.

PEGATEX—PFNonwovens (Non-Woven Fabric)	
Material	Pegatex S anitsat
Lot number	TRZ0A13747
Material number	408243
Weight (gsm)	17

**Table 5:** Frequencies of individual sizes of dust particles on the filter.

Average Frequency Values			
Cutting speed	15 m/min	35 m/min	55 m/min
Feed rate per revolution	0.1 mm/rev.		
Particle size (μm)	Frequency		
1–5	90 ± 12	129 ± 9	154 ± 15
5–10	53 ± 6	53 ± 5	75 ± 7
10–20	17 ± 3	23 ± 4	19 ± 3
30	7 ± 2	8 ± 1	7 ± 1
40	4 ± 1	4 ± 1	4 ± 2
50+	7 ± 1	6 ± 1	8 ± 3
Number	178 ± 13	223 ± 19	267 ± 21

**Table 6:** Average size and frequency of dust particle sizes for different cutting speeds and feed per revolution of 0.1 mm/rev.

Cutting Speed *v_c_* [m/min]	Average Particle Size ± Measurement Uncertainty [μm]	Number of Particles on the Filter
15 m/min	9.01 ± 0.93	178 ± 15
35 m/min	7.79 ± 0.57	224 ± 13
55 m/min	7.30 ± 0.78	266 ± 20

**Table 7:** Percentage ratios of dust particles caught on the filter of the extraction device depend on the cutting speed.

Cutting Speed	15 m/min	35 m/min	55 m/min
Feed per revolution	0.1 mm/rev.		
Holes	Extracted/drilled dust particles (%)		
1–4	28.2	24.5	93.1
6–9	19	31.1	100
11–14	17.6	30.4	99.5
16–19	34.5	59.5	92.8
21–24	19.7	60.5	99.5
26–29	36.5	62.9	96.4
31–34	21.8	78.8	75.7
36–39	31.3	70	81.9
41–44	25.4	45.7	84.6
46–49	27.4	79	99.5
Average value	26.1	54.2	92.5

**Table 8:** Effect of cutting conditions on the wearing of the cutting tool.

Tool Wear VB ± Measurement Uncertainty [μm]			
Cutting speed	15 m/min	35 m/min	55 m/min
Feed per revolution	0.1 mm/rev.		
	19.63 ± 1.92	20.05 ± 1.71	27.13 ± 0.89

**Table 9:** Particle size and frequency as a function of tool wear at increasing cutting speed.

	**1 Drilled Hole**		
	Average frequency values		
Cutting speed	15 m/min	35 m/min	55 m/min
Feed per revolution	0.1 mm/rev.		
Particle size (μm)	frequency		
1–5	28 ± 9	67 ± 9	80 ± 16
5–10	48 ± 12	53 ± 11	73 ± 17
10–20	52 ± 13	68 ± 14	18 ± 3
30	8 ± 3	9 ± 3	8 ± 2
40	6 ± 2	5 ± 2	5 ± 1
50+	7 ± 2	8 ± 3	8 ± 2
	**50 Drilled holes**		
	Average frequency values		
Cutting speed	15 m/min	35 m/min	55 m/min
Feed per revolution	0.1 mm/rev.		
Particle size (μm)	frequency		
1–5	98 ± 10	152 ± 19	174 ± 13
5–10	61 ± 9	92 ± 13	110 ± 9
10–20	20 ± 6	58 ± 10	86 ± 7
30	18 ± 3	25 ± 8	36 ± 6
40	12 ± 4	18 ± 4	19 ± 3
50+	10 ± 2	11 ± 3	15 ± 5

## Data Availability

The original contributions presented in this study are included in the article. Further inquiries can be directed to the corresponding author.
